# An eXplainable Artificial Intelligence analysis of Raman spectra for thyroid cancer diagnosis

**DOI:** 10.1038/s41598-023-43856-7

**Published:** 2023-10-03

**Authors:** Loredana Bellantuono, Raffaele Tommasi, Ester Pantaleo, Martina Verri, Nicola Amoroso, Pierfilippo Crucitti, Michael Di Gioacchino, Filippo Longo, Alfonso Monaco, Anda Mihaela Naciu, Andrea Palermo, Chiara Taffon, Sabina Tangaro, Anna Crescenzi, Armida Sodo, Roberto Bellotti

**Affiliations:** 1 Dipartimento di Biomedicina Traslazionale e Neuroscienze (DiBraiN), Università degli Studi di Bari Aldo Moro, 70124 Bari, Italy; 2 Istituto Nazionale di Fisica Nucleare, Sezione di Bari, 70125 Bari, Italy; 3 Dipartimento Interateneo di Fisica, Università degli Studi di Bari Aldo Moro, 70125 Bari, Italy; 4 Unit of Endocrine Organs and Neuromuscolar Pathology, Fondazione Policlinico Universitario Campus Bio-Medico, 00128 Rome, Italy; 5 Dipartimento di Scienze, Università degli Studi Roma Tre, 00146 Roma, Italy; 6 Dipartimento di Farmacia-Scienze del Farmaco, Università degli Studi di Bari Aldo Moro, 70125 Bari, Italy; 7 Unit of Thoracic Surgery, Fondazione Policlinico Universitario Campus Bio-Medico, 00128 Rome, Italy; 8 Unit of Metabolic Bone and Thyroid Diseases, Fondazione Policlinico Universitario Campus Bio-Medico, 00128 Rome, Italy; 9 Dipartimento di Scienze del Suolo, della Pianta e degli Alimenti, Università degli Studi di Bari Aldo Moro, 70125 Bari, Italy

**Keywords:** Computational science, Applied physics, Biological physics

## Abstract

Raman spectroscopy shows great potential as a diagnostic tool for thyroid cancer due to its ability to detect biochemical changes during cancer development. This technique is particularly valuable because it is non-invasive and label/dye-free. Compared to molecular tests, Raman spectroscopy analyses can more effectively discriminate malignant features, thus reducing unnecessary surgeries. However, one major hurdle to using Raman spectroscopy as a diagnostic tool is the identification of significant patterns and peaks. In this study, we propose a Machine Learning procedure to discriminate healthy/benign versus malignant nodules that produces interpretable results. We collect Raman spectra obtained from histological samples, select a set of peaks with a data-driven and label independent approach and train the algorithms with the relative prominence of the peaks in the selected set. The performance of the considered models, quantified by area under the Receiver Operating Characteristic curve, exceeds 0.9. To enhance the interpretability of the results, we employ eXplainable Artificial Intelligence and compute the contribution of each feature to the prediction of each sample.

## Introduction

Thyroid cancer, consisting in the malignant growth of cells within the thyroid gland, is the most common malignant neoplasia of the endocrine system. Incidence rates, varying worldwide but generally placing it among the ten most prevalent cancers, have increased during the past decades, mostly due to an improvement in diagnostic procedures. The three main types commonly observed of thyroid follicular epithelial cell-derived cancer are papillary thyroid carcinoma (PTC), follicular carcinoma (FC), and the follicular variant of papillary thyroid carcinoma (FV-PTC). PTC is characterized by the presence of papillary structures; it predominantly affects younger individuals and is the most prevalent, accounting for approximately $80\%$ of cases. FC is the second most common type, representing around 10–15$\%$ of cases; it primarily affects older individuals and is more prevalent in areas with iodine deficiency. FV-PTC shares many characteristics with PTC but exhibits a follicular growth pattern, which poses challenges in distinguishing it from FC. Given the significantly high 5-year relative survival rate in early stages, the importance of efficient diagnostic methods cannot be overstated^1^.

Emerging issues in clinical practice include the global increase in detection of thyroid nodules and the consequent rise in the diagnosis of small carcinomas. Other specific challenges are constituted by deciding the extent of surgical treatment and the management of cytologically indeterminate thyroid nodules, and by inter-observer diagnosis variability^2–7^. In addition, during the histological assessment of surgically excised thyroid glands, tumors with a follicular pattern can present diagnostic issues, since the evidence of malignant features, such as capsular or vascular invasion, may not be sufficient; these cases can be classified as follicular tumors of uncertain malignant potential (FT-UMP)^8^, thus leading to a questionable evaluation of the patient’s risk. Over the past fifteen years, there has been a significant increase in the publication of molecular analysis results on thyroid nodule tissue^9,10^. The aim of these studies is to minimize unnecessary surgeries and enhance diagnostic uniformity. A variety of molecular panels and immuno-histochemical tests have been developed for diagnostic and prognostic applications^11^. Although the risk of malignancy linked to various mutational statuses has been suggested as a supplement to the diagnosis of thyroid nodules, only a few of the identified molecular changes have a strong statistical correlation with thyroid cancer diagnosis. Therefore, the positive predictive value of molecular tests remains low^12,13^. Due to these challenges, there is a strong need for the development of a new clinical tool that can accurately detect neoplastic thyroid lesions and improve the differentiation between benign and malignant tumors.

A promising approach to address this issue is provided by Raman spectroscopy (RS), a technique to investigate the properties of matter based on Raman scattering. This phenomenon gives information on the vibrational active modes of molecules through shifts in the scattered light wavelength with respect to the incident one, determined by the difference between the energies of the initial and final vibrational levels. In RS, the observed vibrational fingerprints are associated to the presence and the abundance of specific molecules in a sample, thus providing the ability to distinguish between various chemical states of cells, with specific alterations indicating a possible disease. Since RS allows to detect anomalies in wavelength shift compared to expectations, suggesting the presence of new molecules or modifications in the existing ones, it is widely recognized as a promising approach in identifying cancers^14^. In particular, increasing scientific evidence supports the diagnostic utility of Raman spectra obtained from both cytological and histological samples in the detection of thyroid neoplastic lesions^15–20^. Furthermore, recent findings have demonstrated that RS can serve to support diagnostics as a viable substitute for molecular tests, leading to better management of indeterminate nodules and a reduction in unnecessary surgeries^20^. RS’s capability of identifying specific biochemical changes that occur during oncogenesis, coupled with its non-invasive nature, makes it a highly promising tool to address the current issues in diagnostics. One of the most interesting perspectives for the application of RS to detect thyroid carcinoma is the implementation of an apparatus specifically designed for clinical environments, which would allow to generate spectra from tissues and recognize the fingerprints related to the onset of cancer.

Although the creation of a support system for the diagnosis of thyroid cancer has great potential, its possible use in the clinical setting presents practical and conceptual barriers. These difficulties are related first to the need to correctly understand and interpret the characteristics of the spectra and their link with oncogenesis processes, and then to the inhomogeneity of the diagnostic assessments carried out by different individuals on the basis of a visual inspection of the samples. At present, the utilization of Raman spectra of histological samples in the evaluation of thyroid nodules requires analysis, interpretation and extraction of relevant information by spectroscopists. To overcome these limitations and foster the introduction of RS in the diagnosis of thyroid nodules, it is necessary to develop a reliable and reproducible workflow to translate spectral features, such as peaks and local minima, into a format that can be easily interpreted by medical personnel.

A strategic way to achieve this goal is represented by the paradigm of Artificial Intelligence. In particular, the Machine Learning approach consists in developing algorithms that are trained on a dataset of labelled examples, used as a knowledge base, to identify the characteristic patterns associated with different diagnoses, and subsequently applying the rules thus discovered to the classification of new samples. The crucial advantages of this framework include the possibility of automating the classification workflow, the use of uniform diagnostic criteria for all instances, and the flexibility of the models; the latter are completely data-driven, and therefore have considerable room for improvement thanks to the increasing availability of spectra that can be used in the training phase. Moreover, the implementation of Artificial Intelligence algorithms to classify Raman spectra for diagnostic purposes has already shown great application potential, allowing the automated recognition of fingerprints associated with oncogenesis in different contexts, including cancers of skin^21–23^, digestive system^24–27^, reproductive system^28–30^, brain^31–34^, lung^35,36^, and breast^37–39^. Another particularly interesting case study is the one discussed in Ref.^40^, where Machine Learning models trained on preprocessed Raman spectra in the 400–1800 cm$^{-1}$ range have been used to automatically classify cancerous and normal gastric mucosa, reaching an impressive accuracy of $96.20\%$.

**Figure 1:**
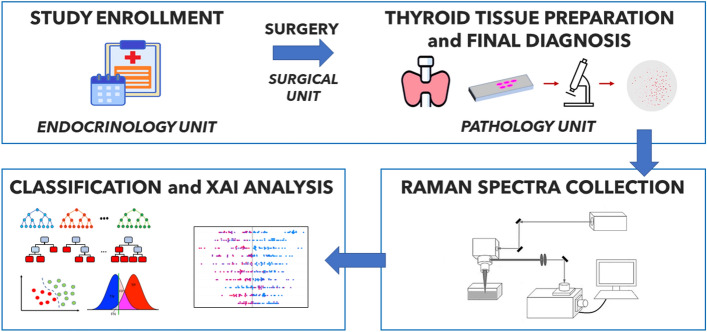
General workflow of the analysis.

In this work, we construct an original dataset of Raman spectra from histological samples, collected in the clinical part of the study, to implement Machine Learning algorithms for the classification of healthy/benign and cancerous samples. The overall workflow followed in the present research is schematized in Fig. 1. The clinical steps (described in detail in the “Methods” section) involve the enrollment of patients with thyroid nodular pathology, a surgery for total thyroidectomy after a cytological diagnosis of malignant, indeterminate, or suspicious lesion, the preparation of tissue samples, and the pathological evaluation. Then, samples are subjected to RS, whose results are used as input for different Machine Learning classification algorithms.

In a previous study^18^, we addressed the problem of thyroid tissue classification with an approach based on clustering analysis. The present work improves the achievements therein in different respects. First, classification in Ref.^18^ was performed in an unsupervised way, evaluating *a posteriori* the differences between samples, as they were captured by a model constructed on the whole dataset. In this article, we bring the potentiality of Artificial Intelligence for Raman spectra analysis to a further step, by constructing *predictive* supervised models, with measurable prediction performances, that allow to classify *new* spectra, not present from the beginning in the training dataset. Moreover, we investigate fingerprints of thyroid cancer by determining, based on rigorous quantitative procedures, the features of Raman spectra that are the most influential on classification outcomes, thus providing a pathway to identify potential biomarkers. For such a purpose, we follow both a global approach, consisting in the Boruta method, in which feature importance is evaluated *a priori* on the training set of spectra, and a local one, based on the eXplainable Artificial Intelligence (XAI) framework. The latter methodology is essential to combine the most relevant requirements of Machine Learning models: (i) informativeness, quantified through performance metrics and uncertainty estimation^41–43^, (ii) generalization, i.e. the reliability of predictions on previously unseen data, and (iii) transparency, which aims to make the decision process as intelligible as possible^44,45^, especially in real-world scenarios^46–52^.

The article is organized as follows: in the “Results” section we show the feature engineering procedure applied to the dataset of Raman spectra, the Artificial Intelligence workflow, consisting in the identification of the optimal Machine Learning classifier and the interpretation of its outcomes trough XAI, and investigate the limitations of the proposed approach when applied to spectra with anomalous properties; in the “Discussion” section we focus on insights and implications of this work and present perspectives for future research; finally, in the “Methods” section, we provide a technical description of the clinical, the spectroscopy and the computational steps of the study.

## Results

The study proposed in the present research consists of three conceptual blocks, highlighted in Fig. 1: (i) the *clinical step*, which includes patient enrollment, surgical excision of thyroid glands and pathological evaluation; (ii) the *spectroscopy step*, in which the Raman spectra associated with each histological sample are obtained; (iii) the *Artificial Intelligence step*, in which we implement a Machine Learning classifier to distinguish the spectra labeled as healthy/benign from those diagnosed with carcinoma, and then we interpret the predictions provided by the model through a XAI analysis. The procedures for carrying out the first two steps are described in detail in the “Methods” section. In the following, instead, we shall focus on the results of the Artificial Intelligence workflow: the classification performance of different Machine Learning algorithms, the fingerprints of the spectra that most influence predictions and, finally, the limitations of the model in the classification of some specific case studies, hereinafter called *ambiguous spectra*, which present anomalous characteristics and are therefore particularly interesting in view of a possible application of the proposed framework in a clinical context.

### Data and feature engineering

The dataset employed in this study comprises 59 Raman spectra obtained from histological samples (tissue slices) excised from the thyroids of individuals with suspected cancer. The samples were examined by the Unit of Endocrine Organs and Neuromuscolar Pathology of Fondazione Policlinico Universitario Campus Bio-Medico, which labeled them as healthy tissues (14 instances), benign adenoma (11), or one of the three most common types of carcinoma: PTC (25), FC (4), and FV-PTC (5). The aims of the analysis are to implement a Machine Learning algorithm capable of classifying Raman spectra, distinguishing healthy or benign nodules (25) from those associated with cancer diagnosis (34), and to identify the main determinants of the model’s predictions using XAI.

The computational workflow starts from a preprocessing stage for the identification of peaks, described in the “Methods” section, in which spectra are interpolated, normalized and fitted with a univariate Gaussian mixture model. Such a preprocessing phase detects 32 peaks in the spectra and assigns a mean Raman shift value $\mu _i$ and standard deviation $\sigma _i$ to each of them. This allows for the creation of an interval $[\mu _i-\sigma _i,\mu _i+\sigma _i]$ for each peak. In order to avoid redundancy, two intervals are initially removed, namely those corresponding to $i=27$ and $i=30$, as they are entirely contained within at least one of the other intervals. Subsequently, we merge pairs of partially overlapping intervals (*i*, *j*) into a single interval $[\min (\mu _i-\sigma _i, \mu _j-\sigma _j),\max (\mu _i+\sigma _i, \mu _j+\sigma _j)]$; this results in the merging of the intervals originally labelled as $i=23$ and $i=24$. The selection process described above gives a set of 29 intervals that do not overlap, which are henceforth identified with new indexes ranging from 1 to 29. The boundaries of these intervals can be found in the Supplementary Table S1. It should be noted that these spectral bands have been identified through a completely data-driven and unsupervised approach from the analysis of the aggregate distribution of all spectra, without any information regarding their diagnostic label.

**Figure 2:**
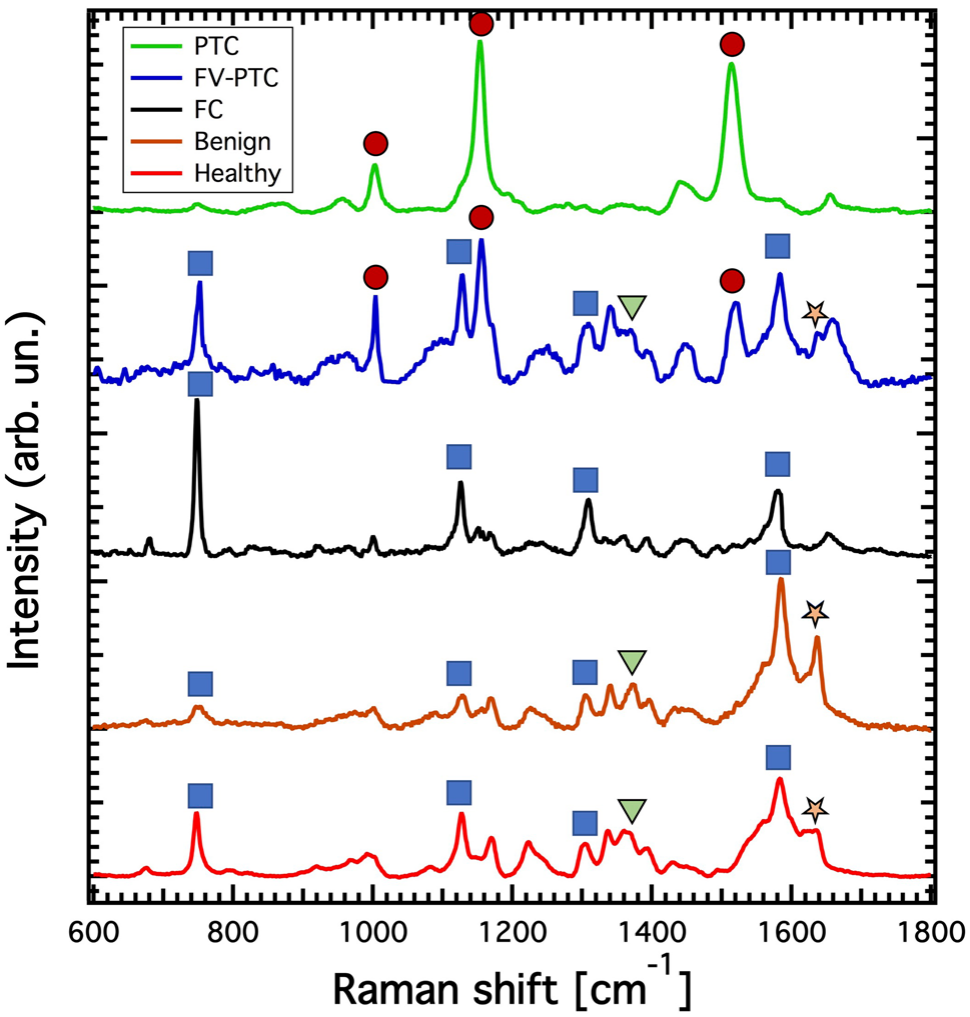
Raman spectra. Typical Raman spectra of the examined thyroid tissues, labelled according to the histology report. Blue squares correspond to the Raman characteristic peaks of reduced cytochrome c, orange stars indicate the spectral lines of oxidised cytochrome c, green triangles the ones of oxidised cytochrome b and the red circles those of carotenoids.

As previously reported in literature, the ability to distinguish between spectra of healthy/benign and carcinoma tissues is attributed to the presence or prominence of various types of spectral lines, including reduced and oxidised cytochrome, and carotenoids^18^. Figure 2 shows the typical structure of the Raman spectra considered in this study, highlighting the characteristic fingerprints of healthy, benign or different types of carcinoma (PTC, FV-PTC, FC) tissues. The relevant characteristic bands in Raman spectra which allow to distinguish healthy/benign and carcinoma tissues are the following, corresponding to specific categories of molecules:

- $$747\; \textrm{cm}^{-1}$$ (reduced cytochrome c)
- $$1003\;\textrm{cm}^{-1}$$ (carotenoids)
- $$1125\;\textrm{cm}^{-1}$$ (reduced cytochrome c)
- $$1155\;\textrm{cm}^{-1}$$ (carotenoids)
- $$1302\;\textrm{cm}^{-1}$$ (reduced cytochrome c)
- $$1376\;\textrm{cm}^{-1}$$ (oxidised cytochrome b)
- $$1516\;\textrm{cm}^{-1}$$ (carotenoids)
- $$1584\;\textrm{cm}^{-1}$$ (reduced cytochrome c)
- $$1638\;\textrm{cm}^{-1}$$ (oxidised cytochrome c).

To compare the spectra in a Machine Learning framework, we create features based on the highest intensity value $P_k$ (prominence) in each of the 29 intervals. These features rely on 812 ratios $P_k/P_{\ell }$ $(k,\ell =1,\dots ,29\,\,k\ne \ell )$ between prominences referred to different intervals. However, the features in the pair $(P_k/P_{\ell },P_{\ell }/P_k)$ are not independent, and it is not clear which one to choose beforehand. In fact, it is not possible to make a selection by comparing prominence values, since they generally change their hierarchy depending on whether the spectrum corresponds to a healthy/benign or cancerous tissue. Additionally, choosing only, e.g., $P_k/P_{\ell }$ with $k<\ell$ is arbitrary. Thus, for each pair $(P_k/P_{\ell },P_{\ell }/P_k)$, we evaluate the distributions of $P_k/P_{\ell }$ and $P_{\ell }/P_k$ on the entire dataset and keep the quantity characterized by the largest ratio between mean value and standard deviation, discarding the other. After this selection process, the number of features is reduced to 406.

**Figure 3:**
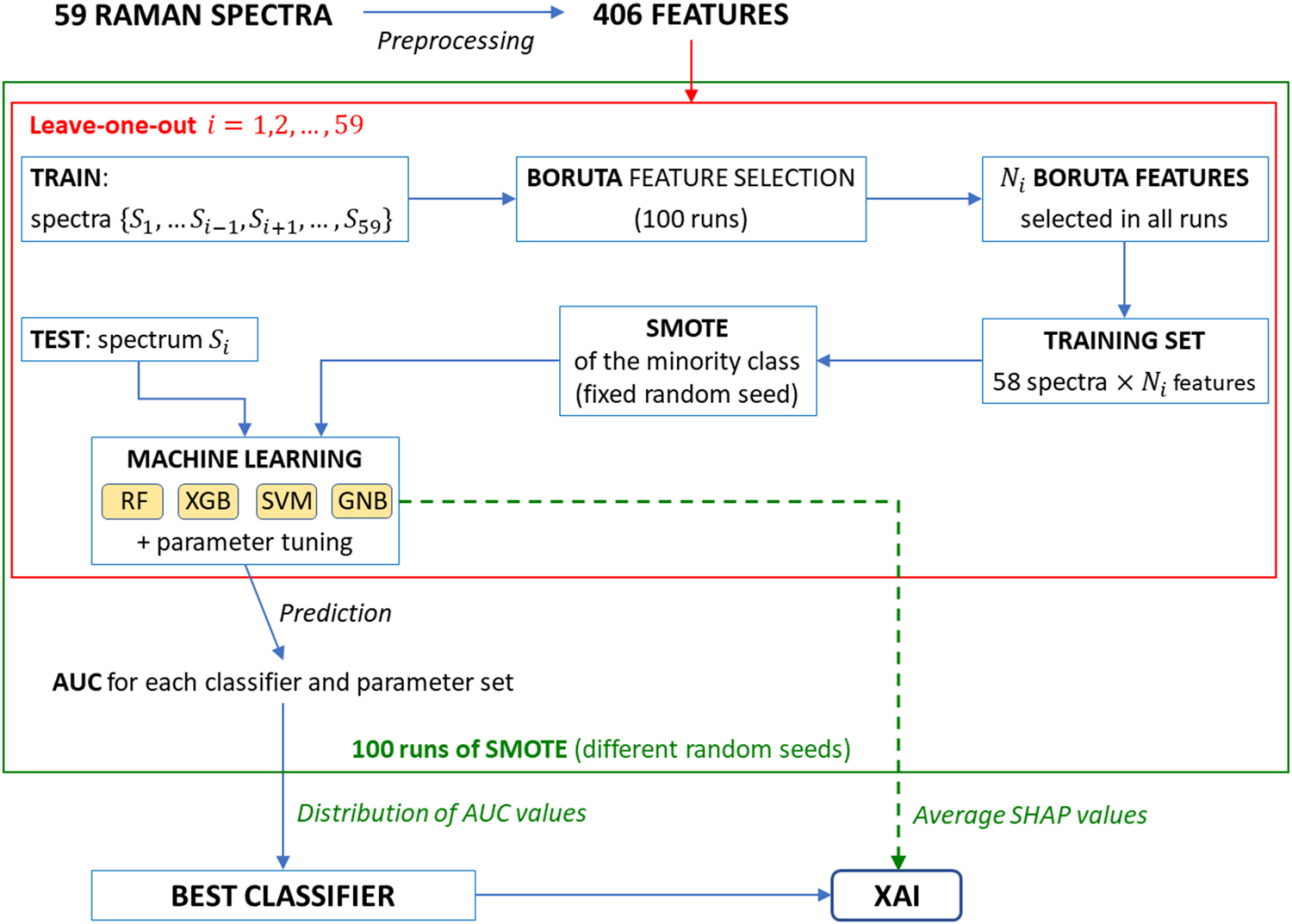
Detailed workflow of the Machine Learning and eXplainable Artificial Intelligence (XAI) analysis. After preprocessing, 100 runs of the synthetic minority over-sampling technique (SMOTE) with different random seeds are executed. In each SMOTE run, a leave-one-out classification is implemented, and in the *i*th leave-one-out iteration (where *i* ranges from 1 to 59) the Boruta algorithm selects $N_i$ relevant features, that are used to construct the training set; then, before implementing different Machine Learning algorithms, SMOTE is applied to oversample the minority class. The classification algorithms employed in this study are random forest (RF), XGBoost (XGB), support vector machine (SVM), and Gaussian Naïve Bayes (GNB). Their performances are quantified by the AUC metrics, which is the area under the receiver operating characteristic (ROC) curve. The impact of features on the prediction for each instance is evaluated through the Shapley (SHAP) values, averaged over all SMOTE runs.

### Artificial intelligence workflow

Figure 3 outlines the Artificial Intelligence procedure that has been implemented in this study to develop a Machine Learning classifier of healthy/benign and carcinoma spectra, and interpret its outcomes through XAI. The workflow contains two nested loops: an outer loop, represented in Fig. 3 as a green rectangle, which consists of multiple executions of the Synthetic Minority Over-sampling TEchnique (SMOTE)^53^, and an inner loop represented by a red rectangle, where a leave-one-out classification procedure is performed. This computational pipeline has been specifically designed to address the case study under consideration, that is based on a dataset of limited size in which the two classes to be distinguished are moderately unbalanced. In particular, the leave-one-out cycle allows to optimize the availability of information to train the algorithm, despite a dataset containing a small number of samples.

During the *i*th leave-one-out trial, where *i* ranges from 1 to 59, spectrum $S_i$ is treated as a test instance, and the feature set is initially reduced using the Boruta feature selection process applied to the 58 remaining training samples $\left\{ S_j\right\} _{j\ne i}$. This algorithm evaluates the importance of each feature by measuring the model performance variation under random shuffling. To ensure control and reproducibility of this process, we conduct 100 Boruta iterations on the dataset of 58 items and 406 features, corresponding to different values of the internal parameter “random_state” ranging from 1 to 100. We select the $N_i$ features that are chosen by *all* the Boruta runs and pass them to the next steps of the Machine Learning workflow depicted in Fig. 3. Then, to compensate imbalances in the training set consisting of 58 spectra and $N_i$ features, we oversample the minority class therein by applying the SMOTE approach. The set thus obtained is used to train a Machine Learning algorithm, which is then validated on the test instance, namely the spectrum $S_i$.

In this workflow we consider the following Machine Learning algorithms: Random Forest, XGBoost, Support Vector Machine and Gaussian Naïve Bayes; for each of them, we explore the internal parameter space in order to identify the optimal configuration. The performance of an algorithm on the entire dataset, i.e., on the whole leave-one-out cycle, is quantified through the area under curve (AUC). This metrics is obtained by evaluating the algorithm performance with varying classification threshold, representing the results as points in a plane where the horizontal and vertical coordinates correspond to the false positive and true positive rates, respectively, and finally computing the area comprised between the receiver operating characteristic (ROC) curve, namely the line connecting the points, and the horizontal axis.

Since the SMOTE algorithm includes random steps, as explained in detail in the “Methods” section, we account for the variability arising from its application by performing 100 leave-one-out cycles for each of the Machine Learning algorithms listed above, keeping their internal parameters fixed. Each trial is associated with a distinct value, ranging from 1 to 100, of the random_state parameter of the SMOTE algorithm that is implemented on the training set. As a result, for each model and each configuration of internal parameters, we acquire a distribution of 100 AUC values, whose median is used as a proxy of the model’s effectiveness. Then, the best classifier is obtained upon comparison among the median AUC values obtained for the different algorithms and internal parameter configurations.

Finally, we inspect the functioning of the best classifier through the XAI approach, by collecting the SHAP values of the different (feature, prediction) pairs, and averaging each of them over the 100 SMOTE runs. SHAP values leverage the interpretability of the classifier as they quantify the impact of the different features on the model’s predictions, revealing connections between Raman spectral properties and diagnoses. In the following subsections we will show the results of the Artificial Intelligence workflow concerning Machine Learning and XAI steps.

#### Machine learning classifier

In this study, we identify as *best classifier* the one with the highest median AUC over the 100 runs of the SMOTE algorithm. If two or more classifiers have the same median AUC, we select the classifier with the lowest interquartile range (IQR) of the AUC values distribution. As highlighted in Fig. 3, we compared the performances of multiple algorithms, namely Random Forest, XGBoost, Support Vector Machine, and Gaussian Naïve Bayes. The parameter space explored for each algorithm is detailed in the “Methods” section.

The best algorithm in terms of AUC for the leave-one-out healthy/benign-versus-cancer tissue classification is Random Forest (median 0.9441, interquartile range 0.0049) with n_estimators$=50$, max_depth$=5$ or 10, and either criterion$=$‘entropy’ or ‘log_loss’ (providing the same results). For definiteness, we will take as a reference henceforth the case with n_estimators$=50$, max_depth$=5$, and criterion$=$‘entropy’. The performances of all the examined algorithms, for the different configurations of their internal parameters, are reported in the Supplementary Information (including Supplementary Table S2). Though the classification outcomes of XGBoost and Support Vector Machine depend on the chosen values of their internal parameters, their median AUC values are instead independent of the specific configuration. For the Gaussian Naïve Bayes classifier, no internal parameter variation has been performed, thus providing a single median AUC value, computed after 100 SMOTE algorithm runs.

**Figure 4:**
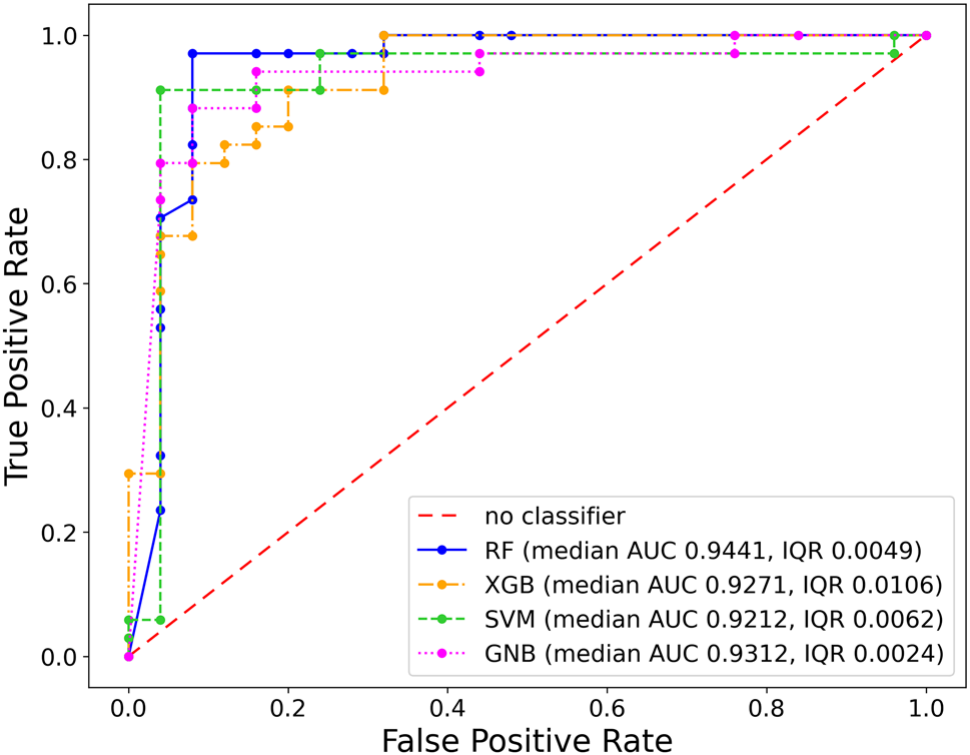
Receiver operating characteristic (ROC) curves for one of the random forest (RF) classifiers that maximize median AUC (n_estimators = 50, max_depth = 5, criterion = ‘entropy’), for XGBoost (XGB) and support vector machine (SVM) algorithms with arbitrary internal parameters, and for the Gaussian Naïve Bayes (GNB) algorithm. Plots referred to XGB and SVM have been obtained in the configurations num_parallel_tree = 100, max_depth = 3, n_jobs = 1, and c = 1, kernel = ‘entropy’, respectively. The True Positive Rate and False Positive Rate coordinates of points in the ROC curves are median values computed over 100 SMOTE runs.

**Figure 5:**
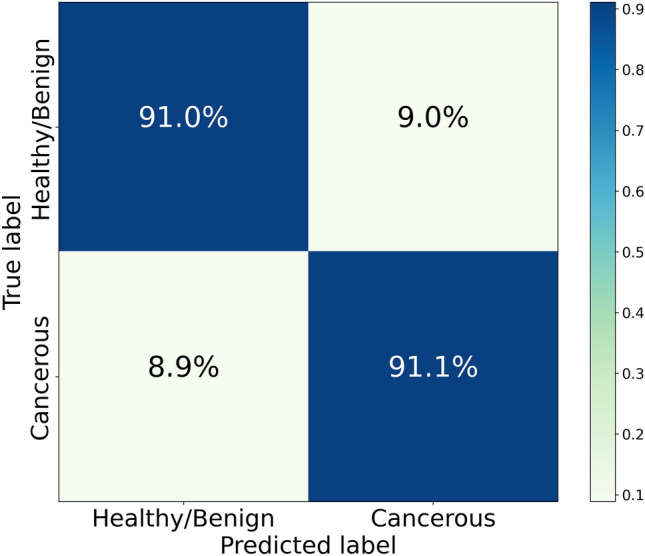
Confusion matrix obtained by collecting the predictions of 100 SMOTE runs, with different random seeds, for a Random Forest model with n_estimators = 50, max_depth = 5, and criterion = ‘entropy’. Such a model provides the best performance in terms of AUC (median 0.9441, interquartile range 0.0049) among the considered ones.

Figure 4 shows the median ROC curves corresponding to the best Random Forest classifier, along with the ones obtained for XGBoost and Support Vector Machine algorithms with arbitrary internal parameters, and for the Gaussian Naïve Bayes one. From the analysis of ROC curves, it is possible to identify, for each model, the optimal classification threshold, as the one that maximizes a specific metric of interest. According to a widely established criterion, we set as the target metric to be maximized $G = \sqrt{\textit{Sensitivity}\cdot \textit{Specificity}}$, namely the geometric mean of sensitivity and specificity, quantifying the balance between these two performance indicators. To determine the optimal classification threshold we choose, for each SMOTE run, the one maximizing *G*. The distribution of classification thresholds found in this way for the best classifier has median 0.5 and IQR 0.065. Figure 5 shows the normalized confusion matrix produced in this optimal case by aggregating the predictions from the 100 runs with varying SMOTE random seeds. The analogous results for the other Machine Learning algorithms are reported in the Supplementary Fig. S1.

Since the presented classification outcomes are averages over 100 SMOTE runs, it is important to evaluate how much the randomness entailed in the artificial oversampling of the minority class in the training set impacts on predictions of test set instances. The stability of classification outcomes provided by the best classifier with threshold 0.5 is satisfactory, with 51 spectra out of 59 that are classified in the same way in all the runs, 3 spectra showing a classification variability below $10\%$, 3 between $10\%$ and $15\%$, and only 2 with higher rates of discrepancy.

Although the proposed model has been optimized for the healthy/benign-versus-cancer tissue classification, it is natural to wonder if its outcomes expressed in terms of prediction probability can be retroactively used to discern diagnostic categories in more detail, also distinguishing Healthy spectra from those generated by benign nodules, as well as the different types of carcinoma. After computing the median prediction probabilities of each spectrum on the 100 SMOTE runs, we aggregate the results based on diagnostic categories, and then compare the respective distributions. Median prediction probabilities corresponding to the different labels are: 0 (with IQR 0.12) for Healthy, 0.04 (with IQR 0.36) for Benign, 0.80 (with IQR 0.45) for FC, 0.92 (with IQR 0.16) for FV-PTC, 0.96 (with IQR 0.22) for PTC.

#### XAI analysis

**Figure 6:**
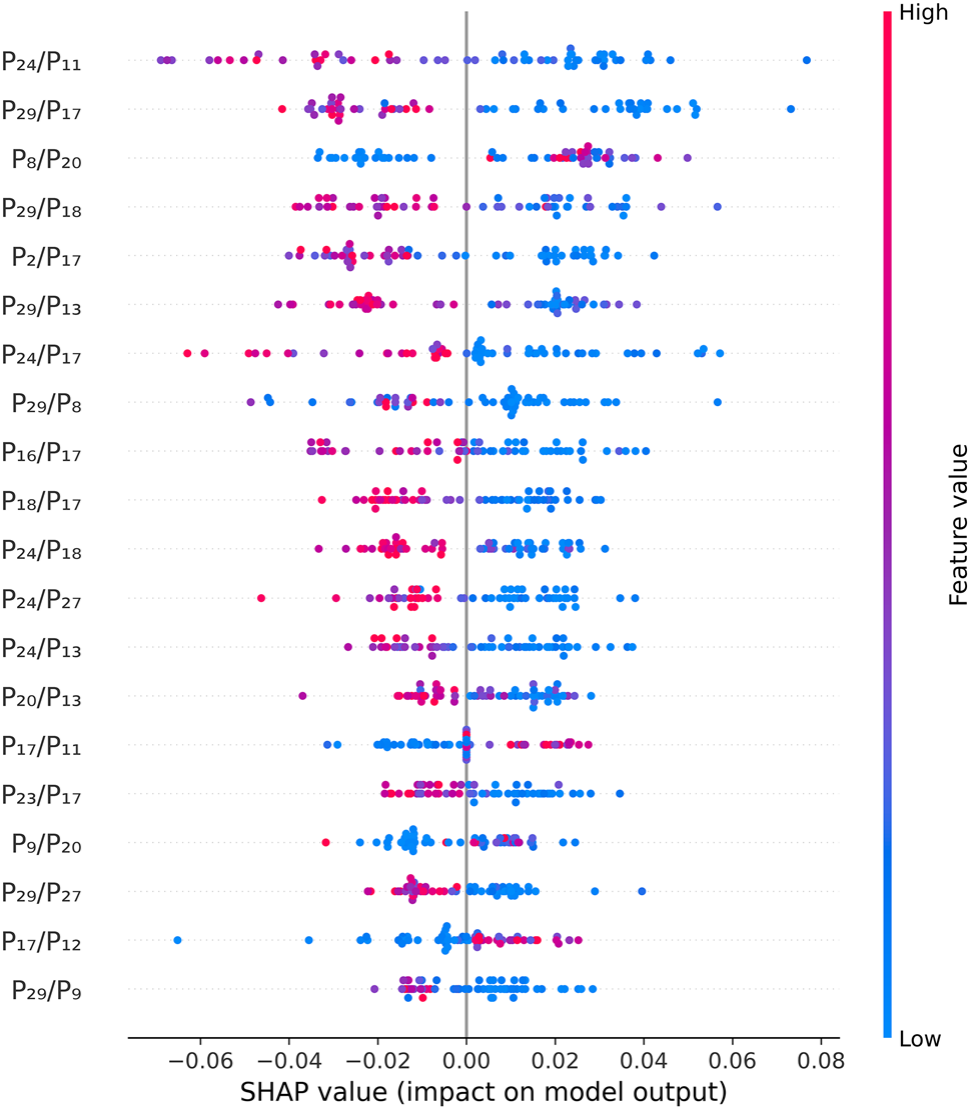
Summary plot of the mean SHAP values, computed on 100 runs of the SMOTE algorithm, with different random seeds, for a Random Forest model with n_estimators = 50, max_depth = 5, and criterion = ‘entropy’.

As a reference for the XAI analysis, we consider the best performing Random Forest classifier, averaging on all the runs the SHAP values associated to the features provided in input by Boruta. A SHAP value 0 is automatically assigned to a feature in a given run, in case it is not selected. In general, SHAP values indicate how a specific feature influences the prediction associated with a given instance. In our analysis, negative and positive SHAP values correspond to a feature’s contribution towards assigning the healthy/benign and cancer labels to an instance, respectively. Each data point in the summary plot depicted in Fig. 6 represents the SHAP value of a particular feature for a specific instance. Higher absolute SHAP values indicate a greater feature relevance in the prediction.

The most influential features, i.e. those with top 20 mean absolute SHAP values on the entire dataset, are the following:

- $P_{24}/P_{11}$, with the interval #24 containing the line $1376\;\textrm{cm}^{-1}$ (oxidised cytochrome b) and the interval #11 not containing lines associated with known categories of molecules; higher values drive the classifier towards the healthy/benign prediction.
- $P_{29}/P_{17}$, with the interval #29 containing the line $1638\;\textrm{cm}^{-1}$ (oxidised cytochrome c) and the interval #17 containing the line $1155\;\textrm{cm}^{-1}$ (carotenoids); higher values drive the classifier towards the healthy/benign prediction.
- $P_{8}/P_{20}$, with the intervals #8 and #20 not containing lines associated with known categories of molecules; higher values drive the classifier mostly towards the cancer prediction.
- $P_{29}/P_{18}$, with the interval #29 containing the line $1638\;\textrm{cm}^{-1}$ (oxidised cytochrome c) and the interval #18 not containing lines associated with known categories of molecules; higher values drive the classifier towards the healthy/benign prediction.
- $P_{2}/P_{17}$, with the interval #2 not containing lines associated with known categories of molecules and the interval #17 containing the line $1155\;\textrm{cm}^{-1}$ (carotenoids); higher values drive the classifier towards the healthy/benign prediction.
- $P_{29}/P_{13}$, with the interval #29 containing the line $1638\;\textrm{cm}^{-1}$ (oxidised cytochrome c) and the interval #13 containing the line $1003\;\textrm{cm}^{-1}$ (carotenoids); higher values drive the classifier towards the healthy/benign prediction.
- $P_{24}/P_{17}$, with the interval #24 containing the line $1376\;\textrm{cm}^{-1}$ (oxidised cytochrome b) and the interval #17 containing the line $1155\;\textrm{cm}^{-1}$ (carotenoids); higher values drive the classifier towards the healthy/benign prediction.
- $P_{29}/P_{8}$, with the interval #29 containing the line $1638\;\textrm{cm}^{-1}$ (oxidised cytochrome c) and the interval #8 not containing lines associated with known categories of molecules; higher values drive the classifier mostly towards the healthy/benign prediction.
- $P_{16}/P_{17}$, with the interval #16 containing the line $1125\;\textrm{cm}^{-1}$ (reduced cytochrome c) and the interval #17 containing the line $1155\;\textrm{cm}^{-1}$ (carotenoids); higher values drive the classifier towards the healthy/benign prediction.
- $P_{18}/P_{17}$, with the interval #18 not containing lines associated with known categories of molecules and the interval #17 containing the line $1155\;\textrm{cm}^{-1}$ (carotenoids); higher values drive the classifier towards the healthy/benign prediction.
- $P_{24}/P_{18}$, with the interval #24 containing the line $1376\;\textrm{cm}^{-1}$ (oxidised cytochrome b) and the interval #18 not containing lines associated with known categories of molecules; higher values drive the classifier towards the healthy/benign prediction.
- $P_{24}/P_{27}$, with the interval #24 containing the line $1376\;\textrm{cm}^{-1}$ (oxidised cytochrome b) and the interval #27 containing the line $1516\;\textrm{cm}^{-1}$ (carotenoids); higher values drive the classifier towards the healthy/benign prediction.
- $P_{24}/P_{13}$, with the interval #24 containing the line $1376\;\textrm{cm}^{-1}$ (oxidised cytochrome b) and the interval #13 containing the line $1003\;\textrm{cm}^{-1}$ (carotenoids); higher values drive the classifier towards the healthy/benign prediction.
- $P_{20}/P_{13}$, with the interval #20 not containing lines associated with known categories of molecules and the interval #13 containing the line $1003\;\textrm{cm}^{-1}$ (carotenoids); higher values drive the classifier towards the healthy/benign prediction.
- $P_{17}/P_{11}$, with the interval #17 containing the line $1155\;\textrm{cm}^{-1}$ (carotenoids) and the interval #11 not containing lines associated with known categories of molecules; higher values drive the classifier towards the cancer prediction.
- $P_{23}/P_{17}$, with the interval #23 not containing lines associated with known categories of molecules and the interval #17 containing the line $1155\;\textrm{cm}^{-1}$ (carotenoids); higher values drive the classifier towards the healthy/benign prediction.
- $P_{9}/P_{20}$, with the intervals #9 and #20 not containing lines associated with known categories of molecules; higher values drive the classifier mostly towards the cancer prediction.
- $P_{29}/P_{27}$, with the interval #29 containing the line $1638\;\textrm{cm}^{-1}$ (oxidised cytochrome c) and the interval #27 containing the line $1516\;\textrm{cm}^{-1}$ (carotenoids); higher values drive the classifier towards the healthy/benign prediction.
- $P_{17}/P_{12}$, with the interval #17 containing the line $1155\;\textrm{cm}^{-1}$ (carotenoids) and the interval #12 not containing lines associated with known categories of molecules; higher values drive the classifier towards the cancer prediction.
- $P_{29}/P_{9}$, with the interval #29 containing the line $1638\;\textrm{cm}^{-1}$ (oxidised cytochrome c) and the interval #9 not containing lines associated with known categories of molecules; higher values drive the classifier towards the healthy/benign prediction.

These results are further analyzed and interpreted in the “Discussion” section.

**Figure 7:**
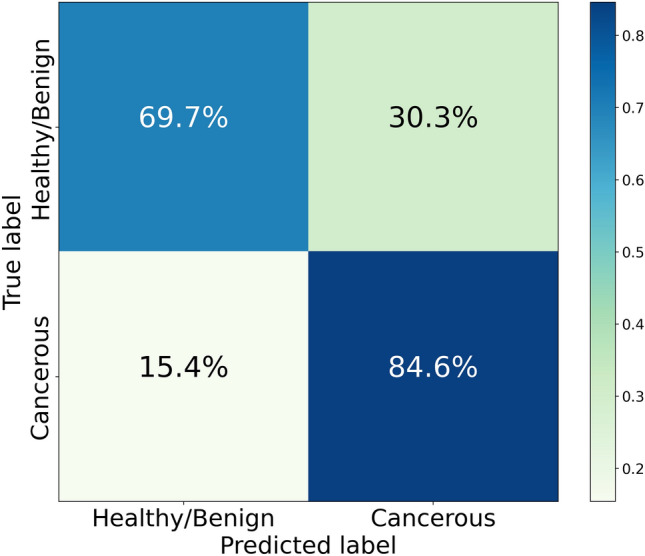
Confusion matrix quantifying the aggregated performance of 100 SMOTE runs of a Random Forest model with n_estimators = 50, max_depth = 5, and criterion = ‘entropy’, applied to all 72 available samples, namely the 59 spectra included in the original dataset and 13 ambiguous spectra. The results are obtained through a two-step process: first, the 59 unambiguous spectra are classified with a leave-one-out procedure, not involving the ambiguous ones; then the 13 ambiguous spectra are classified with the same algorithm trained only on the 59 unambiguous ones.

### Ambiguous samples

Although the proposed model has provided very satisfactory performances in terms of median AUC, and can be straightforwardly interpreted through the XAI approach, it is worth investigating its limitations when applied to the classification of spectra with anomalous properties. For this purpose, we consider 13 additional instances, henceforth called ambiguous samples, whose characteristics differ in many respects from the canonical ones, which would be expected on the basis of their diagnosis.

The 100 runs of the best classifier are applied to all 72 available samples, namely the 59 spectra included in the original dataset and the newly-added 13 ambiguous ones. Such runs correspond to values of the SMOTE random_state internal parameter ranging from 1 to 100. The results presented below are obtained through a two-step process:

- classification of the 59 unambiguous spectra with the same leave-one-out procedure displayed in Fig. 3, performed on the original dataset (that does not comprise the ambiguous samples);
- classification of the 13 ambiguous spectra with the same algorithm, trained on the 59 unambiguous ones.

Predictably, the model performance is reduced in the presence of ambiguous spectra. The resulting AUC distribution from 100 runs has a median of 0.7949 and an interquartile range (IQR) of 0.0135. Figure 7 displays the confusion matrix obtained by combining the predictions from the 100 SMOTE runs on the dataset consisting of 72 spectra. The model erroneously classifies 9 of the 13 ambiguous samples in all runs, one in 99% runs, and one in 75% runs. Of the 9 samples misclassified in all runs

- 2 contain PTC cancerous tissue that is erroneously classified as healthy/benign, since the carotenoid lines are not well visible in their Raman spectra;
- 1 is healthy/benign, but classified as cancerous due to the low visibility of the oxidised cytochrome b line at $1376\,\textrm{cm}^{-1}$;
- 6 are healthy/benign from a histological point of view, but are classified as cancerous due to the presence of mutations, revealed through an immunohistochemical analysis, that determine the presence of carotenoid lines in the spectra. Actually, SHAP values associated with these instances indicate that prominence ratios involving carotenoids have the largest impact on the algorithm decision, as expected since these lines are a characteristic feature of samples associated with a carcinoma diagnosis, particularly PTC and FV-PTC.

The sample misclassified in 99% runs corresponds to a case of FC, difficult to classify due to the absence of carotenoid lines and the scarce representation in the dataset. Finally, the sample misclassified in 75% runs is labelled as healthy/benign, but classified as cancerous due to the low visibility of the oxidised cytochrome b line at $1376\,\textrm{cm}^{-1}$.

## Discussion

In this research work we have developed an Artificial Intelligence workflow capable of interpreting Raman spectra of a particular specimen and providing a highly reliable prediction of the thyroid lesion’s malignancy. A strength of this approach is the fact that the classifier implementation is based on a feature engineering step, which is completely data-driven. Actually, the preprocessing pipeline identifies the interesting intervals from which peak prominences and impactful variables should be extracted in a completely unbiased manner, without using any information about the diagnostic labels associated to the spectra.

Besides being accurate, the predictions provided by the best classifier are also directly interpretable. The results of the XAI analysis indicate a clear pattern consistent with established knowledge: a spectrum with dominant carotenoid lines tends to indicate a cancer diagnosis, while a spectrum with dominant oxidised cytochrome b and c lines is generally associated with a healthy/benign diagnosis. Among the top 20 most impactful features, the prominence referred to the band at $1155\;\textrm{cm}^{-1}$ (interval #17), corresponding to carotenoids, is the most recurrent, as it appears 8 times, 5 of which in the top ten of the same ranking. On the other hand, prominences associated with carotenoid bands at $1003\;\textrm{cm}^{-1}$ (interval #13) and $1516\;\textrm{cm}^{-1}$ (interval #27) appear few times and have a lower impact on prediction. This finding is noteworthy because it suggests a potentially different importance hierarchy for cancer biomarkers. Further investigation is needed to determine if this hierarchy holds when analyzing a larger dataset. The most impactful features also include the two lines at $1376\;\textrm{cm}^{-1}$ (interval #24) and $1638\;\textrm{cm}^{-1}$ (interval #29) corresponding to oxidised cytochromes b and c, respectively. It is worth mentioning that only one reduced cytochrome c line, specifically that at $1125\;\textrm{cm}^{-1}$ (interval #16), appears in one of the influential model features reported in Fig. 6. This is due to the fact that reduced cytochrome c lines are not able to definitely differentiate between healthy/benign and cancer-related spectra, as they are prominent in spectra with both FC and FV-PTC tumor diagnoses, while being undetectable in the case of PTC.

The present study was conducted with a dataset of limited size and characterized by a subject imbalance between the healthy/benign and cancer categories. The Machine Learning pipeline, shown in Fig. 3, employs two nested iterative procedures, SMOTE and leave-one-out, both designed to tackle datasets with such problematic features, providing satisfactory performances. Nonetheless, the limited size of the dataset and the scarce representativeness of FC and FV-PTC subclasses prevented us to implement a more targeted Artificial Intelligence workflow, that could distinguish between the different thyroid carcinoma categories. On the other hand, the performed analysis provided encouraging indications that the proposed algorithm could accomplish this kind of classification, when trained on a larger dataset. Actually, in a previous study^18^, a clustering linkage algorithm highlighted a ranking of the different types of carcinoma tissue, based on their similarity with the healthy/benign one; the extremes of such hierarchy are FC (most similar) and PTC (least similar). Remarkably, the same ordering seems to emerge also in the present research work, by comparing the distributions of the median prediction probabilities, computed on the 100 SMOTE runs, referred to spectra belonging to different diagnostic categories. The corroboration of such a finding would benefit from a larger dataset containing enough representatives of each category, which we plan to investigate in future research to further validate our workflow and its potential applicability in a clinical setting.

To identify the limitations of the proposed model, we tested its ability to classify spectra that have anomalous characteristics, inconsistent with those expected on the basis of their diagnostic label. The application of the optimal algorithm to the 6 instances with mutations is noteworthy. These samples, misclassified in 100% of SMOTE runs, are considered healthy based on histological analysis, but an immunohistochemical test reveals the presence of mutations resulting in peaks corresponding to carotenoids. It is possible that these samples were excised before the onset of the disease. However, medical opinions on this matter are divided, and it is not universally accepted that tissues exhibiting these characteristics will inevitably progress to cancer. In such cases, it is generally recommended to operate on the patient. While the classifier may make formal assignment errors on such histological samples, it highlights an interesting class of tissue from a clinical perspective.

The results of the study suggest that the use of Artificial Intelligence for the healthy/benign-versus-cancer classification of histological samples can lay the foundations for promising innovations in the clinical field, allowing the development of new devices to support diagnosis. In fact, the proposed workflow has the potential to enable fast and nearly real-time lesion classification, standardize Raman spectra interpretation and reduce costs associated with patient management, especially if its application is extended to samples that can be acquired with less invasive procedures, such as fine needle aspiration.

## Methods

### Study enrollment, clinical evaluation and tissue preparation

The enrollment phase, managed by the Unit of Metabolic Bone and Thyroid Diseases of Fondazione Policlinico Universitario Campus Bio-Medico, lasted from January 2018 to October 2021. All patients were submitted to US scan of thyroid gland and neck area, performed with a frequency range of 10–12 MHz on a MyLab 50 (Esaote, Genova, Italy) by 2 experienced endocrinologists at the Metabolic Bone and Thyroid disordes Unit. The observed nodules were classified according to ACR TI-RADS risk stratification criteria^54^. In doubtful cases the endocrinologists conducted a separate session to reach a unified consensus. Patients with clinical or US characteristics indicating the need to perform fine needle aspiration according to the literature^55^, were asked to sign the informed consent to participate in the study. Only patients who underwent surgery (total thyroidectomy) after a cytological diagnosis of indeterminate, suspicious or malignant nodule, in according to the international guidelines^56^, were definitely enrolled in this study. The thyroidectomies were carried out at the Unit of Thoracic Surgery of the aforementioned Institution. Study population included 54 subjects (34 females, 20 males) affected by thyroid nodular pathology, with age distribution centered at 46.3 years, with a 11.2 years standard deviation. Specimens removed during surgery were promptly submitted unfixed to the Unit of Endocrine Organs and Neuromuscolar Pathology of the same Institution in an properly labelled container. Here, after evaluating the gross anatomy of the samples, tissues slices of about 1 cm $\times$ 1 cm $\times$ 3 mm were cut, including both healthy and neoplastic areas, avoiding surgical margins. Tissue slices were snap frozen in the cold plate of a cryostat. A 5$\upmu$m section was stained with haematoxylin/eosin to confirm the presence of healthy and cancerous tissues, and then 4 consecutive cryostatic sections were cut at a thickness of 30 $\upmu$m, collected on separate slides and stored at a temperature of $-20\,^{\circ }$C. The Raman analysis was exclusively performed on these frozen unfixed samples. For definitive histology, the residual slices were defrosted, fixed in formalin, and embedded in paraffin along with the paired surgical samples. The final diagnosis was made in agreement with current edition of WHO classification of endocrine tumours^57^. Paraffin sections from neoplastic areas in each patient were used for immunohistochemical analysis of Galectin3 (Gene Tex), CD56 (Agilent), and HBME1 (Agilent) using an automated immunostainer (Omnis, Agilent)^18^.

### Raman measurements

We acquired Raman spectra using a Renishaw InVia Micro-Raman spectrometer and a solid-state diode laser source at 532 nm with a nominal output power of about 100 mW for excitation. In our experimental arrangement, we focused the laser beam onto the sample (unfixed 30 $\upmu \textrm{m}$ section) and gathered the back-scattered unpolarized intensity using either a Leica 50$\times$ LWD objective or an Olympus 100$\times$ objective mounted on a Leica DM2700 M confocal microscope. The investigation areas of cancerous and healthy tissues were defined by the correspondence of the subsequent sections with that characterized with hematoxylin-eosin, described above. The laser beam could be focused on the sample in a spot of a few microns in diameter, thus allowing for the separation of the signal contribution originating from the cells under investigation . Neutral-density filters were used to reduce the power of the laser beam incident on the sample to prevent photo-damage. Our setup employs a holographic edge filter to ensure high-contrast rejection of the elastically scattered light. A 1800 grooves/mm diffraction grating is utilized to disperse the Raman inelastic scattering contribution and a $1024 \times 256$ pixels, Peltier-cooled, CCD camera is used to detect the scattered light. We collected punctual spectra by utilizing the extended scan mode across the 100–3600 $\textrm{cm}^{-1}$ Raman-shift range, with a spectral resolution of approximately $1 \; \textrm{cm}^{-1}$. For each sample, we carried out five measurements at selected points, with five scans acquired for each point. The cumulative integration time for each point was almost 50 seconds. The Renishaw Wire software was used to collect the raw spectra and to perform data reduction, such as background and fluorescence subtraction.

### Preprocessing of the spectra and feature extraction

The spectra are preprocessed using the following steps. Firstly, all spectra are interpolated to a Raman shift grid with equal spacing of $1 \; \textrm{cm}^{-1}$. Next, each spectrum is normalized to have a sum of one (area under curve equals to one), and then cubic spline smoothing is performed. Peak detection is then carried out on each preprocessed spectrum, and the resulting local maxima are collected from all spectra. A univariate Gaussian mixture model with unequal variance is used to fit the distribution of the local maxima across the 59 samples, and the optimal model is selected based on the Bayesian Information Criterion (BIC)^58^. We use R (version 4.2.2) packages gsignal^59^ (version 0.3-5) to find peaks and mclust^60^ (version 6.0.0) to fit a Gaussian mixture model to the histogram of the local maxima.

### Boruta feature importance

In order to mitigate the effects of noise and data redundancy, we utilize a wrapper method for feature selection based on the Boruta framework^61^. This procedure identifies only those features that are uncorrelated with each other and significantly improve the performance of the machine learning algorithm. The Boruta feature selection tool is based on a supervised learning Random Forest algorithm, of which it exploits the founding concept: randomizing the training samples and perturbing the system helps to mitigate the negative impact of random fluctuations and correlations in the learning model.

In the Boruta framework, the original set of features is expanded by adding *shadow* features, which are constructed by randomly shuffling the values of each original indicator. This augmented dataset is then used to train a Random Forest algorithm, which is capable of making predictions and evaluating the importance of both the original and shadow features. Boruta selects features that, within the dataset, provide statistically more accurate predictions than those obtainable by replacing them with their corresponding shadow counterparts, after conducting a series of independent shuffling operations. As a result, the competition among features in Boruta does not require the use of an arbitrary importance threshold to determine which variables are relevant, as is often necessary in traditional feature selection techniques.

In this work, we implement in each Boruta run a Random Forest algorithm (RandomForestClassifier function), with n_jobs$=-1$, max_depth$=5$, and other parameters set to default values. The internal Boruta parameters include “estimator” set to “estimator_forest”, “n_estimators” set to “auto”, and “max_iter” set to 500. We use Python (version 3.9) package boruta^62^ (version 0.3) to implement the Boruta algorithm.

### SMOTE algorithm

Imbalanced classification refers to the task of building predictive models on classification datasets where one class has significantly fewer examples than the other. The main difficulty of working with imbalanced datasets is that standard machine learning techniques often ignore the minority class, leading to poor performance on it. A common solution to this problem is to oversample the minority class examples, which involves duplicating them in the training dataset prior to model fitting. Although this can balance the class distribution, it does not add any new information to the model. A more effective strategy than duplicating minority class examples is to generate new instances by synthesizing them from the samples already existing in the minority class. This approach, known as Synthetic Minority Oversampling TEchnique (SMOTE), involves a type of data augmentation for the minority class^53,63^. To create synthetic examples, SMOTE first selects a random instance *a* from the minority class and identifies its *k* nearest neighbors within the minority class. A synthetic instance is then generated by selecting one of the *k* nearest neighbors *b* at random and connecting *a* and *b* to form a line segment in the feature space. Hence, the synthetic instance is created as a convex combination of the two selected examples *a* and *b*, at a randomly selected point between them.

In this study, minority class oversampling is performed within each leave-one-out iteration, as described in Fig. 3. In particular, we implement the SMOTE function, setting the number of nearest neighbors to $k=10$, and we control its randomness by fixing the internal parameter random_state. The stability of the classifier outcomes with respect to the oversampling procedure is assessed by executing 100 SMOTE runs, for random seed values between 1 and 100, and analyzing the distribution of performance indicators of Machine Learning algorithms. We use Python (version 3.9) package imbalanced-learn^64^ (version 0.10.1) to implement the SMOTE procedure.

### Random forest

A random forest (RF) algorithm consists in an ensemble of decision trees obtained by resampling the training dataset with repetitions (bootstrapping)^65^. The randomization procedure on the features in the training phase ensures that the mutual correlation between RF trees is low. Decision trees provide independent predictions about each observation, and then the results of all trees are combined together, by either averaging in the case of regression, or majority voting in the case of classification. The key features of RF algorithms are their simple tunability, the small number of parameters to set, the robustness with respect to overfitting, the possibility to evaluate feature importance during the training phase, and the unbiased estimate of the generalization error. In this study, to determine the best performance of the healthy/benign-versus-cancer classification in the leave-one-out mode, the following Random Forest parameters are varied:

- n_estimators $$\in \{25,50,100\}$$,
- criterion $\in \{$‘gini’,‘entropy’,‘log_loss’$\}$,
- max_depth $$\in \{3,5,10\}$$.

The best result is obtained with the parameter choice n_estimators$=50$, max_depth$=5$ or 10, and either ‘entropy’ or ‘log_loss’ criteria, providing median AUC equal to 0.9441, with interquartile range 0.0049. The RF algorithm is implemented in the Python (version 3.9) package scikit-learn^66^ (version 1.1.2).

### XGBoost

The XGBoost algorithm utilizes an ensemble of decision trees, which are trained through an iterative gradient boosting process. This involves addressing critical points that arise in the decision trees at each step by the subsequent trees. The XGBoost algorithm tackles the problem of missing values by using sparsity-aware split finding, which exploits the data sparsity patterns in a unified way and learns the optimal direction to take in case of a missing feature required for the split^67^. To determine the best performance of healthy/benign-versus-cancer classification in the leave-one-out mode, the following XGBoost parameters are varied

- num_parallel_tree $$\in \{25,50,100\}$$,
- max_depth $$\in \{3,5,10\}$$,
- n_jobs $$\in \{1,10,100\}$$,

while keeping importance_type set to “gain” mode and other parameters set to default values. All the configurations provide median AUC equal to 0.9271, with interquartile range 0.0106. The XGBoost algorithm is implemented in the Python (version 3.9) package xgboost^68^ (version 1.6.2).

### Support vector machine

Support Vector Machine (SVM) is based on determining the optimal boundary between two or more classes in the data space by minimizing a loss function called Hinge Loss, to which a penalty term is added^69^. In this algorithm, only a limited number of input observations, called support vectors, play a relevant role to identify the boundary between classes. The SVM algorithm proceeds iteratively, keeping misclassified occurrences as support vectors that contribute to the loss proportionally to their distance from the boundary. In such a way, the loss depends only on a subset of the input observations, allowing for an efficient estimate of the optimal parameters. To determine the best performance of healthy/benign-versus-cancer classification in the leave-one-out mode, the following SVM parameters are varied:

- c $$\in \{0.5,1,2,3\}$$,
- kernel $\in \{$‘linear’,‘poly’,‘rbf’,‘sigmoid’$\}$.

All the configurations provide median AUC equal to 0.9212, with interquartile range 0.0062. The SVM algorithm is implemented in the Python (version 3.9) package scikit-learn^66^ (version 1.1.2).

### Gaussian Naïve Bayes

Gaussian Naïve Bayes (GNB) is a generative classification algorithm, that constructs full statistical Gaussian models involving both feature values and output labels, using the Bayes rule^70^. The term “naïve” refers to the forced assumption that all pairs of features are conditionally independent, given the output labels. GNB is built easily and with no complicated iterative parameter estimation required. In classification problems, the model evaluates the conditional probabilities that a given instance corresponds to the different classes, and then returns as prediction the label that maximizes such probability. For the healthy/benign-versus-cancer classification, the algorithm, with no internal parameter variation, provides median AUC equal to 0.9312, with interquartile range 0.0024. The GNB algorithm is implemented in the Python (version 3.9) package scikit-learn^66^ (version 1.1.2).

### eXplainable Artificial Intelligence

The eXplainable Artificial Intelligence (XAI) framework encompasses a range of techniques that share a unified view, which incorporates informativeness, uncertainty estimation, generalization, and transparency. In this study, the SHAP local explanation algorithm is utilized to identify the importance of features for classifying healthy/benign and carcinoma histological samples.

The SHAP algorithm is a local, model-agnostic post-hoc explainer that is based on the concept of Shapley (SHAP) values, derived from cooperative game theory^71,72^. It learns local interpretable linear models for each sample, focusing on the contributions of each feature to the prediction of that sample. To calculate the SHAP value for a given feature, the algorithm evaluates the difference between the model output’s prediction with and without that particular feature, considering all possible subsets of features. Therefore, the model must be retrained on all feature subsets *F* of the complete set *S* of features ($F \subseteq S$). If $f_{x}(F)$ is the model’s prediction for instance *x* given a subset *F* that does not include, e.g., the *j*th feature, and $f_{x}(F \cup {j})$ is the prediction when the *j*th feature is added, the marginal contribution provided by the *j*th feature can be computed as the difference $f_{x}(F \cup {j})-f_{x}(F)$. The SHAP value of the *j*th feature for the instance *x* is then calculated by adding it to all possible subsets:

1 $$\begin{aligned} SHAP_j(x) = \sum _{F\subseteq S - \{j\}} \frac{|F|!(|S|-|F|-1)!}{|S|!} \left[ f_{x}(F \cup {j})-f_{x}(F)\right] , \end{aligned}$$

where |*F*|! represents the number of permutations of features in the subset *F*, $(|S|-|F|-1)!$ represents the number of permutations of features in the subset $S-(F\cup \{j\})$, and |*S*|! is the total number of feature permutations^71^. The SHAP value computation is implemented in the Python (version 3.9) package shap^73^ (version 0.41.0).

### Ethics statement

The study protocol adhered to the Declaration of Helsinki and to the International Conference on Harmonization Good Clinical Practice and received approval by the Ethical Committee of the “Fondazione Policlinico Universitario Campus Bio-Medico” (UCBM) (prot. 33.15 TS ComEt CBM and 31/19 PAR ComEt CBM from 26th July 2019). All participants granted informed consent. Enrolled patients were recorded in a codified file with an anonymous ID code, which was registered in the software database of the Endocrine Organs and Neuromuscolar Pathology Unit of the UCBM.

### Supplementary Information

Supplementary Information.

## Data Availability

The dataset used and analyzed during the current study and computer code are available from the corresponding author on reasonable request.
