# Supplementary material for: An eXplainable Artificial Intelligence analysis of Raman spectra for thyroid cancer diagnosis
Source: Sci Rep. 2023 Oct 3;13:16590. doi: 10.1038/s41598-023-43856-7 (PMC10547772; doi:10.1038/s41598-023-43856-7)
Supplement: Supplementary file 1 — Supplementary Information. [file 41598_2023_43856_MOESM1_ESM.pdf]

SUPPLEMENTARY INFORMATION
An eXplainable Artificial Intelligence analysis of Raman spectra for thyroid cancer diagnosis

Loredana Bellantuono[1,2], Raffaele Tommasi[1], Ester Pantaleo[2,3], Martina Verri[4,5], Nicola Amoroso[2,6], Pierfilippo Crucitti[7], Michael Di Gioacchino[5,*], Filippo Longo[7], Alfonso Monaco[2,3], Anda Mihaela Naciu[8], Andrea Palermo[8], Chiara Taffon[4], Sabina Tangaro[2,9], Anna Crescenzi[4], Armida Sodo[5], and Roberto Bellotti[2,3]

[1]Università degli Studi di Bari Aldo Moro, Dipartimento di Biomedicina Traslazionale e Neuroscienze (DiBraiN), Bari, I-70124, Italy
[2]Istituto Nazionale di Fisica Nucleare, Sezione di Bari, Bari, I-70125, Italy
[3]Università degli Studi di Bari Aldo Moro, Dipartimento Interateneo di Fisica, Bari, I-70125, Italy
[4]Unit of Endocrine Organs and Neuromuscolar Pathology, Fondazione Policlinico Universitario Campus Bio-Medico, Rome, I-00128, Italy
[5]Università degli Studi Roma Tre, Dipartimento di Scienze, Roma, I-00146, Italy
[6]Università degli Studi di Bari Aldo Moro, Dipartimento di Farmacia-Scienze del Farmaco, Bari, I-70125, Italy
[7]Unit of Thoracic Surgery, Fondazione Policlinico Universitario Campus Bio-Medico, Rome, I-00128, Italy
[8]Unit of Metabolic Bone and Thyroid Diseases, Fondazione Policlinico Universitario Campus Bio-Medico, Rome, I-00128, Italy
[9]Università degli Studi di Bari Aldo Moro, Dipartimento di Scienze del Suolo, della Pianta e degli Alimenti, Bari, I-70125, Italy
[*]michael.digioacchino@uniroma3.it

ABSTRACT

This Supplementary Information document consists of two sections, containing additional materials that are not included in the main text. The first section reports the 29 intervals obtained from the preprocessing pipeline. The second one is focused on the healthy/benign-vs-cancer classification performances of the different Machine Learning algorithms, for the explored configurations of their parameter spaces.

Selected intervals

Table S1 displays the boundaries and identifiers of 29 distinct intervals in the Raman spectra, which were carefully selected to avoid overlap. These intervals were utilized to assess the peak prominence values, from which our set of features was created.

Performance comparison of Machine Learning algorithms

We present a comprehensive analysis of the performance of all Machine Learning algorithms included in our study: Random Forest (RF), eXtreme Gradient Boosting (XGB), Support Vector Machine (SVM) and Gaussian Naïve Bayes (GNB). We examine the performance of these algorithms by exploring their internal parameter space. In particular, for a given model and a fixed configuration of its internal parameters, we assess the median Area Under Curve (AUC) from a distribution of 100 values obtained by modifying the random_state of the SMOTE algorithm used on the training set. Table S2 displays the results for the RF algorithms, which are slightly sensitive to the parameter choices. Instead, the results obtained for XGB and SVM do not depend on the chosen settings, while GNB has no internal parameter variation; in these cases, the results are

- median AUC 0.9271 and interquartile range 0.0106 for XGB,
- median AUC 0.9212 and interquartile range 0.0062 for SVM,
- median AUC 0.9312 and interquartile range 0.0024 for GNB.

Interval ID	Lower bound (cm^{-1})	Upper bound (cm^{-1})
1	616.78	652.25
2	669.18	674.83
3	687.48	698.95
4	714.87	721.90
5	745.77	750.14
6	776.26	805.89
7	821.99	831.17
8	848.93	855.68
9	868.54	881.28
10	914.81	949.94
11	957.94	961.01
12	962.08	970.68
13	996.83	1003.60
14	1028.71	1046.63
15	1077.27	1098.03
16	1122.99	1127.41
17	1151.18	1155.83
18	1164.00	1169.88
19	1188.23	1200.32
20	1220.92	1250.26
21	1276.20	1284.35
22	1302.98	1311.06
23	1335.59	1341.87
24	1356.56	1383.21
25	1389.05	1395.33
26	1398.48	1476.25
27	1512.49	1517.84
28	1551.78	1611.36
29	1628.54	1637.00

Table S1. Boundaries and identifiers of the 29 non-overlapping selected intervals in the Raman spectra, providing the basis for constructing features.

We report for completeness in Figure S1 the confusion matrices describing the classification performances of XGB, SVM and GNB algorithms, considering that in the first two cases performances do not depend on parameters. Finally, the distributions of optimal classification thresholds, obtained by maximizing the geometric mean of sensitivity and specificity for each SMOTE run, provide the following median values and interquartile ranges:

- median threshold 0.4845 and interquartile range 0.3494 for XGB,
- median threshold 0.5242 and interquartile range 0.0114 for SVM,
- median threshold 0.9990 and interquartile range 0.0028 for GNB.

The wider threshold variability highlights that the implemented XGB algorithms are highly sensitive to the SMOTE random seed, which reflects in poorer average performances reported in the related confusion matrix.

n_estimators = 25	**max_depth** = 3	**max_depth** = 5	**max_depth** = 10
**criterion** = **'gini'**	0.9388 (0.0071)	0.9441 (0.0053)	0.9441 (0.0053)
**criterion** = **'entropy'**	0.9424 (0.0054)	0.9441 (0.0059)	0.9441 (0.0060)
**criterion** = **'log_loss'**	0.9424 (0.0054)	0.9441 (0.0059)	0.9441 (0.0060)
n_estimators = 50	**max_depth** = 3	**max_depth** = 5	**max_depth** = 10
**criterion** = **'gini'**	0.9394 (0.0065)	0.9418 (0.0056)	0.9418 (0.0059)
**criterion** = **'entropy'**	0.9424 (0.0054)	0.9441 (0.0049)	0.9441 (0.0049)
**criterion** = **'log_loss'**	0.9424 (0.0054)	0.9441 (0.0049)	0.9441 (0.0049)
n_estimators = 100	**max_depth** = 3	**max_depth** = 5	**max_depth** = 10
**criterion** = **'gini'**	0.9365 (0.0047)	0.9400 (0.0053)	0.9400 (0.0053)
**criterion** = **'entropy'**	0.9365 (0.0049)	0.9406 (0.0049)	0.9406 (0.0050)
**criterion** = **'log_loss'**	0.9365 (0.0049)	0.9406 (0.0049)	0.9406 (0.0050)

Table S2. Median AUC on the classification outcomes corresponding to 100 runs of the SMOTE algorithm, computed by utilizing a Random Forest algorithm with different internal parameters. The numbers in brackets represent the interquartile ranges of the distributions.

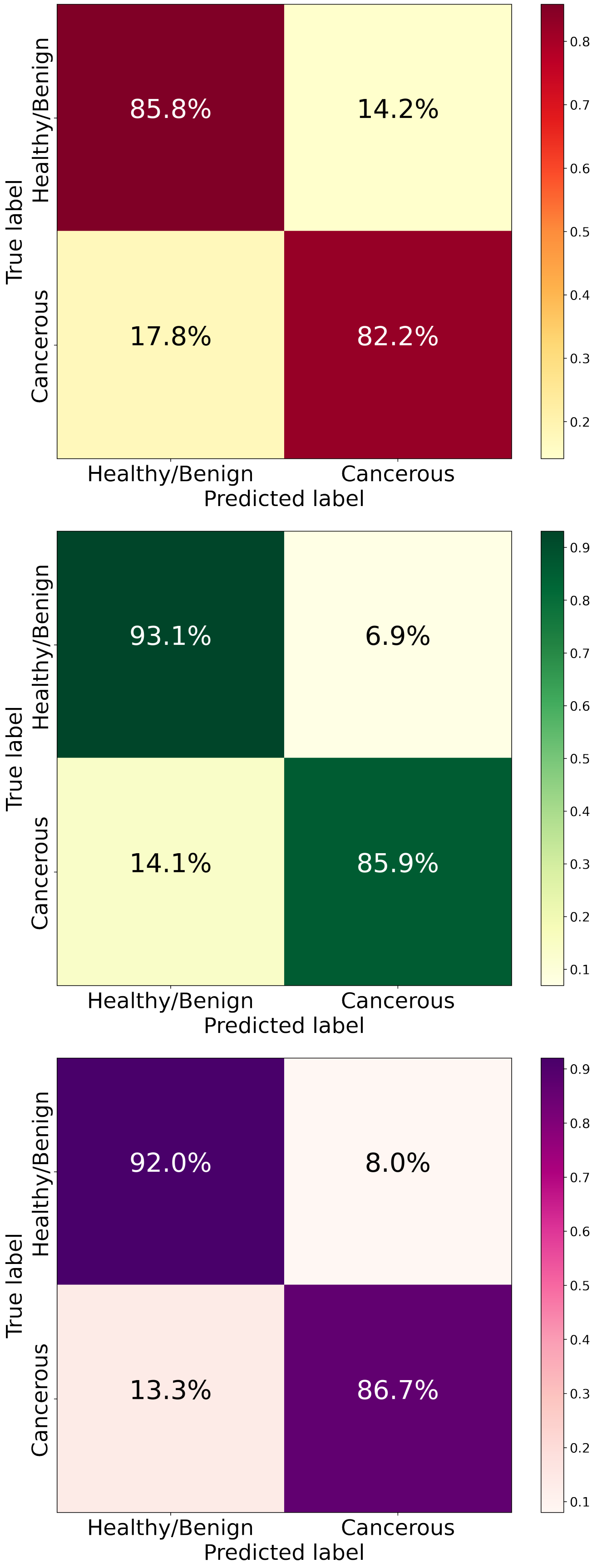

Figure S1. Confusion matrices describing the classification performances of XGB (top panel), SVM (central panel) and GNB (bottom panel) algorithms.
